# Perceptions of Elephants’ Labor and Autonomy in Zoos

**DOI:** 10.3390/ani15233410

**Published:** 2025-11-26

**Authors:** Angela M. Lacinak

**Affiliations:** Social and Political Sciences, Philosophy, and Anthropology, University of Exeter, Exeter EX4 4PY, UK; am932@exeter.ac.uk

**Keywords:** elephants, zoos, visitors, autonomy, animal labor, animal emotions, animal welfare, anthrozoology

## Abstract

Many modern zoos maintain charismatic megafauna, such as elephants, as their iconic residents, as these animals have been shown to hold the zoogoers’ gaze more adeptly than demurer animals. This study sought to determine zoo supporters’ perceptions of elephants’ emotions and welfare under conditions in which elephants receive rewards and demonstrate behaviors within elephant–caregiver interactions in a zoo to generate key themes associated with those perceptions and to compare those perceptions to zoo professionals’ through the lens of The Five Domains animal welfare assessment model. This qualitative study employed multiple methodological strategies, including surveys, autoethnography, interviews, and welfare assessments. The article explores two themes that were generated regarding participants’ perceptions: elephants’ autonomy and participation in labor. The discourse of elephant labor in a zoo is timely, as animal labor among domestic animals is a growing topic in social sciences research. Elephants’ welfare was perceived as largely positive, though there was a small number of participants who felt the elephants did not enjoy their labor. Participants’ perceptions were mostly aligned with zoo professionals’ perceptions. As this is the first article to discuss zoo animal labor, it makes a unique contribution to the current literature and may have applications to other species’ labor within zoos.

## Simple Summary

Elephants are among the most popular attractions in zoos and are frequently included in training demonstrations for public viewing. This study explored zoo supporters’ perceptions of elephants’ welfare and emotions during these training interactions with a focus on the behaviors requested of the elephants and the rewards they received from their caregivers. Research data were collected through online surveys, interviews, animal welfare assessments, and the author’s historic knowledge based on a career that spans three decades of work in zoos. This article focuses on two themes that were generated during analysis of the data: elephants’ autonomy or ability to make decisions and their participation in labor/work within zoos. Elephants’ welfare was perceived by participants as mostly positive, though there were minimal references to the animals not enjoying the work they undertook. This research is the first to explore the concept of animal labor for zoo animals and therefore makes a significant contribution to the current literature and may have applications for other animals’ labor within zoos.

## 1. Introduction

Many modern zoos maintain charismatic megafauna, such as lions, tigers, great apes, and elephants, as their iconic residents. There is good reason for this, as these non-human animals (henceforth animals) have been shown to hold zoogoers’ gaze more adeptly than demurer animals [1]. As elephants are frequently sought by zoo visitors, perceptions of their welfare and emotions are of importance to zoos as they strive to engage their patrons in intimate encounters, often involving elephant–caregiver interactions (ECIs). Teaching animals in zoos using a symbiotic ethical ethos and with skilled human staff is a noteworthy aspect of the bolstered welfare zoos’ resident animals experience today. According to Fernandez and Martin [2], “reward-based husbandry training practices” are one of the two “cornerstones of the modern zoo” (p. 531). Frequently, in modern zoos, animals are taught to voluntarily allow medical procedures, such as vaccination administration as a component of annual wellness exams and ultrasonography to monitor fetal development or reproductive viability, and to accomplish daily husbandry practices, such as shifting from one space to another so that caregivers may clean or provide enrichment (the second cornerstone of modern zoos, according to Fernandez and Martin [2]).

Behavioral conditioning or teaching of animals is also frequently utilized by zoos to provide visitors with experiences or demonstrations. Furthermore, these interactions have been evidenced to provide opportunities for novel incorporation of welfare assessments by visitors that align with those of zoo professionals (e.g., [3,4]). This level of visitor engagement may serve to encourage repeat visitation and makes transparent the welfare-assessing process that many zoos employ (e.g., those that attain accreditation through organizations such as the Association of Zoos and Aquariums [AZA]). Though not all animals in zoos are incorporated into dynamic training regimes, the complex requirements of managing elephants have resulted in a mandatory behavioral repertoire consisting of 15 behaviors to attain accreditation from the AZA, according to The Accreditation Standards and Related Policies [5]. This article, which centers on ECIs, explores the exchanges between the individuals as collegial, that is, the elephants and caregivers work (labor) together symbiotically in pursuit of a shared goal and the elephants make autonomous choices in doing so.

This research is an anthrozoological undertaking, as it explores the entanglements of three key stakeholders that make up the community within zoos’ boundaries: zoo visitors, caregivers, and the animal residents. Specific terminology is incorporated throughout this article to bring the elephants to the fore of the research. For example, in lieu of the more traditional label of human–animal interactions (HAIs), this article uses elephant–caregiver interactions (ECIs). This places emphasis on the importance of the elephants involved in the research. Additionally, the term caregiver is utilized in lieu of the more traditional label of zookeeper, in acknowledgement of the education and skillset required of most zoological professionals [6].

### 1.1. Animal Labor Theory

The results of this article introduce the concept of animal labor, a unique finding for zoo-related discourse. Therefore, a review of Animal Labor Theory is warranted. Karl Marx, credited with founding modern labor theories, believed animals incapable of the complexities required of consciously engaging in cooperative work activities [7]. The idea that animals are just tools for human use, however, is shifting, particularly in the social sciences. Kendra Coulter put forth the concepts of humane jobs for animals and interspecies solidarity [8,9,10]. According to Coulter [10], humane jobs are “jobs that are good for both people and animals, and that are underscored by multispecies respect” (p. 32).

Like the work represented in this article, Coulter [10] takes a pragmatic approach to much of her work in pursuit of affecting real-world changes (p. 32). She attests to believing that jobs for some animals can be humane, such as for horses and dogs. However, she posits that the only animals appropriate for humane work are those previously domesticated. She states, “a foundational requirement of potential humane jobs for animals is that only already domesticated species and individual animals are ethically appropriate for consideration… wild and/or captive animals should not be working for people” [11] (p. 35).

Coulter’s stance on this divide between what may be appropriate for domesticated animals and those in zoo environments regarding their ability to perform humane labor is problematic for several reasons. When considering ethical jobs for animals, one must consider how the animals are taught the skills that they execute. Though there are exceptions (e.g., animals that are taught using tools of coercion), many modern accredited zoos employ positive reinforcement training (PRT) protocols for teaching animals that do not rely on electrified, pronged, or pinch collars, mouth bits, head harnesses, or whips (as many dog and horse trainers do with animals that Coulter attests are suited for labor). This should be evaluated on a case-by-case basis, as the philosophical approach to modifying behavior of any species, domesticated or not, is reliant on the ethics of the human trainer. Furthermore, based on my extensive experience of more than three decades working with zoos, their caregivers, and animals, I argue that zoo animals’ “jobs” are typically less stressful for the animals than, say, mounted horses that serve police officers in cities or military dogs who are taught to run toward danger with their soldier handlers.

Zoo animals are largely employed as what could be labeled “passive performers.” Most zoo animals would not have a memory of anything beyond their lived zoo experience, as most were born in zoos [12]. Their daily activities include resting, eating, mating, playing, exploring enrichment, navigating social dynamics, interacting with their caregivers, etc., while zoo visitors observe. Many are taught to participate in their own medical care, so they receive training regularly, though the amount of time spent on these activities in zoos likely pales in comparison to the “working” time per day of service, military, or police animals. Most zoo animals are trained for minutes, not hours, per day (personal experience). It is possible, however, that zoo animals’ welfare would increase with more training in the form of ethical labor (though this is currently anecdotal and would require additional research).

To fully consider the impact of this research on the elephants themselves, obtaining elephants’ consent to participate in this study was regarded as a requisite. Though most studies incorporate an informed consent statement, the symbiotic pragmatic ethics that permeates this research required a more thorough look at elephants’ consent to engage in the activities required for this study. Though “there is a scarcity of information guiding the design of ethics protocols for research with animals, and fundamental gaps in the procedural structures of ethics reviews,” there are calls to incorporate “less speciesist” parameters into the ethics approval process for research involving participant animals [13] (pp. 172–173). Following recommendations by Van Patter and Blattner [13], this research incorporated their three principles for ethical standards: non-maleficence, beneficence, and voluntary participation. Table 1 illustrates these recommendations, their functional definitions, and the practical application of each principle as it relates to this study.

### 1.2. Aims

This research aimed to (i) determine current and potential zoo visitors’ perceptions of elephants’ emotions and welfare under key conditions of ECIs (rewards and behaviors), (ii) understand why those perceptions were formed, (iii) assess actual elephant welfare under the target conditions of ECIs, and iv) formulate pragmatic forward-facing actions that have the potential to benefit zoo visitors, elephants, and elephant caregivers.

## 2. Materials and Methods

This predominantly qualitative case study employed multiple methodological strategies, including surveys, autoethnography, interviews, and welfare assessments. A Deweyan pragmatism conceptual framework, following Kelly and Cordiero [14] and Lacinak [3,4], was employed. The bulk of the web-hosted survey consisted of open-ended qualitative questions incorporating a video elicitation approach. The surveys required observation of short video clips and then listing participants’ generated descriptors or justifications, a modified free choice profiling (FCP) approach. Wassermann et al. [15] conducted a study on perceptions of marine mammal parks using a photo elicitation approach and found the method less biased than those that queried participants without visual media. As my research involved active interactions between elephants and humans, video clips were selected to provide better context for those reciprocal exchanges rather than the static glimpses provided by photographs.

Though photo elicitation and video elicitation methods may introduce bias (as the photos and videos are choreographed or selected by the researcher), Wassermann et al. [15] attest that “photo elicitation reduces reliance on pre scripted questions and responses, and seems to effectively reduce other forms of bias” (p. 1). Video elicitation in data collection is also an effective methodology. According to Griffin [16], who explored the use of videos as a data collection tool, it has been utilized in a variety of social research, “but its application within tourism and leisure studies is comparatively sparse” (p. 2184). Following Greco et al. [17], who researched elephant management in North American zoos utilizing online surveys and purposive sampling, data are excluded from partially completed surveys.

Electronic surveys have advantages (flexibility, reducing interviewer bias as the surveyor is not present, and convenience for the researcher) but also several disadvantages. Among the challenges of e-surveys are potential poor response rates and confidentiality of the participants [18]. To address confidentiality concerns, a GDPR-compliant survey-hosting site was utilized (rather than social-media-developed surveys, such as through Facebook), and participant identifiers are not discussed. Following recommendations on survey organization by Ruel et al. [19], this survey comprised three sections: an introduction that described the nature of the research, a video elicitation segment that utilized open-ended questions, and, lastly, a demographics segment.

Respondents were asked to list descriptors of their choosing to characterize elephants’ emotional states. This approach follows the work of Wassermann et al. [15], who used open-ended questions and a photo elicitation methodology to capture public perceptions of marine mammal attractions that included cetaceans (*Orcinus orca* and *Tursiops truncatus*). The decision to allow respondents to provide their own terms was made to obtain the most accurate impression possible, as opposed to having participants select from the researcher’s provided estimations, which may or may not match the respondents’ perceptions.

FCP was introduced by Wemelsfelder et al. [20] in a study where naïve observers conducted Qualitative Behavior Assessments (QBA) of pigs (*Sus scrofa*) and found that observers were able to reliably interpret the animals’ welfare states based on assessments of the pigs’ body language or movements. They defined FCP, an experimental process at the time, as giving the observer “complete freedom to choose their own descriptive terms” [20] (p. 209). In 2016, Minero et al. used a video elicitation methodology with students to contrast the reliability of FCP with fixed-list (FL) methodologies in QBAs. They found that students under FCP and FL paradigms similarly perceived the pigs’ movements, further validating the approach [21].

During the online surveys, participants were asked to state the word or phrase that they believed best described how the elephant in the clip they had just observed felt (in clips A–F that showcased rewards given to and behaviors requested of elephants). They were next asked to explain why they believed the elephant felt as he/she did. Participants were then asked to identify which clip they felt reflected the best welfare by dragging and dropping the video clips into positions 1–3, where 1 = highest level of welfare and 3 = lowest level of welfare.

This article also incorporated autoethnography and interviews as additional data, drawing on the author’s three decades of work within zoos with a specialization in animal (including elephant) behavior and purposively selected animal experts. Autoethnographic reflections and interview data are utilized in comparison to zoo visitors’ perceptions (i.e., similarities or disparities) and to better understand the context under examination. Semi-structured interviews were conducted in person. The interview with Burns, a Curator who oversaw the elephant team at the time of this research, was conducted on 5 December 2019 on the grounds of Zoo Tampa. The interview with de Waal was conducted on the grounds of the Palm Beach Zoo and Conservation Society on 1 February 2020. Interviews were recorded on an iPad Pro via the Voice Memos app and then transcribed using a transcription app, Transcribe, for initial processing. Transcripts were then manually edited for verbatim accuracy.

Another avenue in which this article incorporates the views and contributions of individuals with specialized skills or training is through welfare assessments of the animals participating in the ECIs represented within the case study by the author and Zoo Tampa’s caregivers and veterinarians. According to Binding et al. [22] (p. 166), “the predominant framework for understanding animal welfare in the international zoo community is the Five Domains model” that was developed and evolved by Mellor et al. [23,24,25,26,27,28,29] and incorporated into welfare guidelines produced by the World Association of Zoos and Aquariums [30].

This article represents a selection of the data collected for a larger research endeavor. The larger study explored messaging delivered to visitors in addition to the rewards and behaviors discussed within this article (truncated for publication) and represents one case study of four that were conducted.

### 2.1. Sampling

Participants were purposively selected based on their level of support of zoos, as this case study sought to examine the perceptions of zoo visitors or supporters. The survey was available to participants from 31 July to 7 September 2019. Participants were primarily recruited using social media posts (social media announcements with a link to the survey were posted on the author’s three Facebook accounts) with encouragement to share the survey link, a snowball or referral technique of purposive sampling. This form of web-based survey sampling was undertaken in a case study that explored grief in animal care workers [31]. Similarly, the researchers of that study used the same Qualtrics survey software platform, excluded surveys that were not fully completed, and recruited participants primarily “through social media blurbs” [31] (p. 34). Given the similarities in methodology between their study and this study, their snowball sampling methodology was a good fit for this case study, as well. Participants were further recruited through referrals from professors at four universities/colleges (the universities/colleges were Eckerd College in St. Petersburgh, Florida, The University of Florida in Gainesville, Florida, Beacon College in Lakeland, Florida, and The University of Pennsylvania in Philadelphia, Pennsylvania) who teach within animal-studies-related programs. The same criteria of zoo support applied to these participants, and the criteria of snowball or referral techniques under the umbrella of purposive sampling are the best distinction for this pool of informants.

It is noted that the social media pages upon which this survey was shared were largely animal-related and therefore not representative of the larger public. It is an acknowledged limitation of purposive sampling that the results cannot be generalized to the larger public [32]. However, as the goal was to capture the perceptions of zoos’ visitors and/or supporters, this sampling methodology was appropriately suited to this study.

A total of 706 participants engaged with the survey. The survey was completed in full (every question was answered) by 154 participants (22%). As this article specifically sought to obtain the perceptions of zoo visitors and zoo supporters, participants who rated that they were unsupportive of zoos (indicated by selection of 1 or 2 on a Likert scale of 1–5, where 1 = very unsupportive and 5 = very supportive) were also removed from the data. This resulted in the removal of data for a further 7 participants, yielding 147 viable participant surveys or 21% of the total number of participants who engaged on any level.

Given the open-ended format of the queries and the time requirement to observe the video clips, a level of respondent fatigue or burden was anticipated [19]. Electronic surveys have been documented to produce poor response rates, with online surveys averaging a 29% completion rate (compared to 50% for in-person surveys) [33]. This average indicates that the viable responses from the participant pool for this case study were slightly below average. The time invested by participants in the survey varied widely, but most were completed within 8–25 min.

Most of the participants were educated females (almost 80% were females, and three-quarters held a 4-year degree or higher). Participants ranged in age from 20s to 80s, representing a wide age demographic. More than six in ten had some experience working with animals (either paid or unpaid; not specific regarding species).

### 2.2. Setting

All video clips were recorded at Zoo Tampa in Florida, USA. Two Zoo Tampa elephant caregivers and two African elephants participated in the recorded sessions for the video elicitation methodology. The caregivers, Mike and Sue, were experienced elephant professionals in management positions. Sdudla was an adult male elephant. Ellie was an adult female elephant. Zoo Tampa manages their elephant herd in a protected contact setting, meaning the caregivers and elephants do not share the same space. Therefore, all video clips depicted elephants behind containment barriers.

As an animal care professional accustomed to working in protected contact settings (and understanding the ethical implications of free contact work with elephants), it did not occur to me at the time of data collection that containment barriers in the video clips might impact observers’ perceptions or to offer an explanation of this during the survey process. This is an acknowledged limitation, as exhibit design has been shown to affect zoo visitors’ perceptions. For example, one study found that unnatural exhibits resulted in visitors describing zoos in negative terms [34]. Though the elephants at Zoo Tampa have naturalistic looking exhibits, the video clips were recorded in their off-exhibit paddocks to facilitate ease of interactions with their caregivers, and, therefore, large metal bollards were visible (an unnatural aesthetic).

Another study attested that a naturalistic ape exhibit contributed to increased concern for the primates and a “reduced utilitarian attitude” compared to more traditional habitats [35] (p. 435). However, in a study that explored zoo visitors’ perceptions of elephants’ welfare, scientists found that “observing elephants engaged in a variety of species-typical behaviors and having an up-close experience was significantly correlated to visitors having a positive emotional response” [36] (p. 1). Therefore, participants observing elephants engaged in activities (though the proximity was virtual) may have countered any negative responses due to the visible containment.

### 2.3. Video Clips’ Content

**Figure 1:**
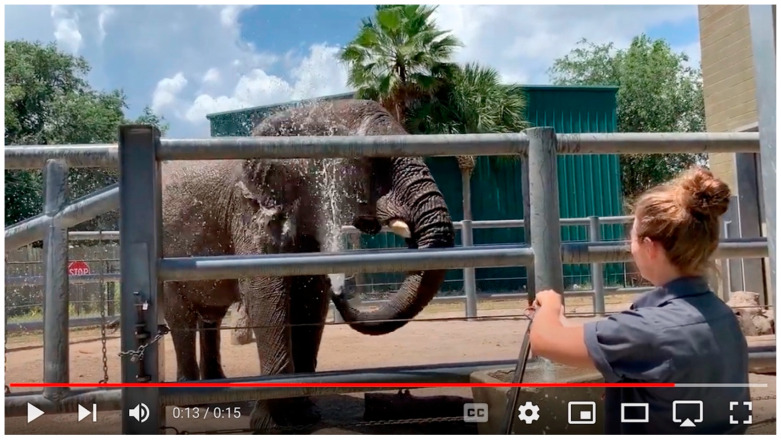
Clip C, Sdudla splashes himself with water provided by Sue (source: author).

The six video clips represented in this article were recorded with an iPad Pro or an iPhone^XR^. Table 2 provides a reference for the clips, including the emotional descriptors offered by participants for the elephants under conditions in each category (discussed fully in Section 3.1). Three clips represented rewards given to elephants by their caregivers: produce, taction in the form of petting and scratches, and water (for play or bathing; Figure 1). These specific rewards were chosen due to their contrasting nature and because food and tactile stimulation are the most frequent reinforcers utilized by the Zoo Tampa team with their elephants [37] (personal observation). The third option of water for play or bathing fell into a less frequently offered category that included items for interaction, such as toys. Furthermore, providing various forms of water for enrichment is common in American zoos and includes pools, sprinklers, waterfalls, and streams [17].

**Figure 2:**
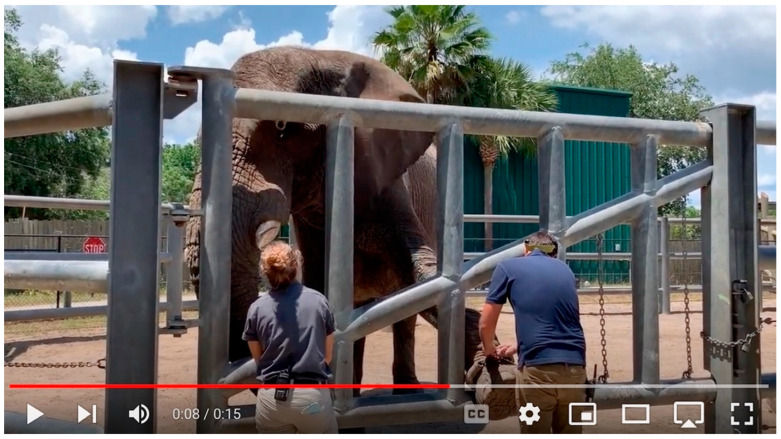
Clip F, Sdudla presents his left foot for anklet placement (source: author).

Three clips depicted behaviors the elephants demonstrated at the request of their caregivers: climbing the bollard fencing with the elephant’s front legs, resulting in weight being shifted to the hind legs (a behavior referred to as “grandstand”), painting a canvas, and allowing a caregiver to secure a metal anklet onto a front foot (Figure 2). The grandstand selection was made to demonstrate a high energy expenditure behavior that visitors might associate with a performance or trick behavior (that one might observe at a more theatrical venue, such as a circus). An enrichment-oriented behavior (painting on a canvas) was chosen to potentially indicate cognitive stimulation. The medical or restraint behavior (anklet placement) was selected for its ambiguity, as it can be interpreted in a multitude of ways, including dominance, excellent health care, or benign activity tracking (personal experience and external feedback from reviewers provided as the survey was being finalized).

Each clip was 15 s in duration. The clips’ duration was selected to allow enough time for an impression to be formed while also providing adequate context for the parameter demonstrated but not so much time that multiple perceptions might compete with one another. Video clips as short as 10 s in length have been utilized in zoo survey research, as demonstrated by Razal and Miller [38], who investigated zoo visitors’ perceptions of natural versus unnatural environmental enrichment in animal enclosures. Razal and Miller’s [38] work demonstrated that video clips need not be lengthy to allow participants to form impressions of animal welfare (among other parameters). The clips were uploaded to YouTube in unlisted format, i.e., they were not visible without the associated link. Clips were muted so that participants focused on the elephants’ and caregivers’ interactions without sonic information. The clips maintained static positions within the survey (the order was not randomized).

### 2.4. Data Analysis

Reflexive Thematic Analysis (RTA) was used to explore the *why* responses to survey questions (e.g., why the respondent perceived the elephant as happy while being petted by the caregiver and/or why the respondent felt the clip of the elephant having an anklet placed on his/her leg reflected a higher level of welfare than standing on his/her hind legs or painting a canvas). This analysis was inductive in that “the analysis is located within, and coding and theme development are driven by, the data content” (as opposed to a deductive orientation, where pre-established theories shape the analytical process [39] (p. 10). The six-step process followed recommendations by Braun and Clarke of familiarization with the raw data, coding, generation of initial themes, further development of themes in consideration of the full dataset, naming and defining of themes, and writing up the analysis [39,40,41]. Data were organized and analyzed manually using Microsoft Excel. For transparency, Table 3 illustrates the RTA process for this study. A similar RTA methodology was incorporated into two other zoo-related studies involving visitor perceptions [3,4].

The RTA process involves multiple layers of reviewing the dataset in relation to theme generation, resulting in revisions throughout the process [39]. As an example of this, the theme of “autonomy” was conceived in early stages of theme generation as “cooperation.” Upon further reflection of participant quotes and meaning making analysis, however, it became clear that this cooperation was an expression of the elephants’ autonomy. This organic evaluative process was continued until the themes reflected well the intention of the participants and supported the aims of the study [39,42]. This is described by Braun and Clarke [39] as preferable in RTA analysis to attempts at data saturation. They attest that the quality of coding in RTA “stems not from consensus between coders, but from depth of engagement with the data, and situated, reflexive interpretation... this process-based, and organic, evolving orientation to coding makes saturation (especially conceptualised as information redundancy) difficult to align” [42] (p. 9).

## 3. Results and Discussion

### 3.1. Emotional Descriptors and Welfare States

The emotional descriptors that participants proffered regarding their perceptions of the elephants as a result of the video elicitation methodology were primarily positive and are listed in Table 2. It is of interest that all elephants under conditions of receiving rewards from a caregiver, regardless of the type of reinforcement, were perceived as happy by a large portion of respondents. It is also noteworthy that all most-mentioned emotional descriptors, apart from hot, warm, and hungry, reflect positive affective states. The descriptors of hot, warm, and hungry do not necessarily reflect poor affective states. They must be considered alongside the respondents’ justification for the descriptor proffered for insight into how the descriptors were intended for interpretation.

Elephants’ emotional states were also interpreted to be largely positive under conditions of elephants executing behaviors, with all most-mentioned descriptors reflecting positive affective states. Given that elephants under conditions of receiving reinforcement were perceived as largely happy (as one of the most-mentioned emotional descriptors), it is noteworthy that the only behavior condition that led to a perception of the elephant as happy was when the elephant painted on a canvas. The remaining two behavioral conditions did receive mentions of the elephants as happy, but by a very small portion of the respondents (less than 5%). In fact, there were no emotional descriptors that aligned as most mentioned for all three clips under the behavior context (see Table 2).

Under conditions of the elephants receiving rewards from their caregivers, respondents selected Clip C where the elephant sprayed water (provided by the caregiver) on his body as reflecting the highest level of welfare of the three (Table 2, Figure 1) and Clip A where the elephant received food as the lowest. Perhaps the participants’ perceptions aligned with de Waal’s (in interview, 2020) assertion that food received from a trainer might not be a highly effective reinforcer [43]. He stated, “the elephants eat so much and so constantly, that the little tidbit like an apple is no big deal for them; I’m not sure food is the top of the priority list as long as they get enough” [43] (lines 123–125).

This raises an interesting point regarding providing edible rewards to an animal the size of an elephant. Burns (in interview, 2019) countered, however, that his experience led him to believe that strategic offerings of food could indeed please, and therefore reinforce, the elephants [44]. He stated, “they are highly reinforced by some of the primaries like larger items, whether it is a big old flake of alfalfa [a type of grass] or a melon... you’ll see an elephant’s eyes light up when you show them a big watermelon in the summertime or show them a pumpkin in the fall” [44] (lines 235–238).

Both de Waal and Burns make salient points in this instance. I have witnessed trainers tossing small diet components (such as an apple or a slice of sweet potato) into an elephant’s mouth with very little detectible reaction from the animal. They accept the items, but there is no visible excitement or appreciation for the caregiver’s offerings. To Burn’s point, however, I have witnessed elephants display anticipatory body language, such as wide eyes, shuffling of feet, reaching of trunks, and vocalizing, when trainers approach with food that is not available daily (and therefore possesses novelty).

Under conditions of the elephants demonstrating behaviors, respondents selected Clip F, where the elephant allowed an anklet to be secured around his leg, as reflecting the highest level of welfare of the three (Table 2, Figure 2). The elephant painting was perceived as only slightly higher in welfare than the grandstand behavior. Unlike Clip C, the anklet behavior received the least mentions of happy as the elephant’s emotional state (two participants). However, it is apparent from the descriptors mentioned by most respondents that they interpreted this behavior as reflective of a trusting (the most mentioned emotional descriptor) relationship between the elephant and the caregiver.

### 3.2. Generated Themes

Many of the respondents used consistent descriptors and justifications for their perceptions of elephants’ emotions, resulting in four generated themes across the video clips. These themes were ECRs, enjoyment of the activity, autonomy, and elephant labor. As is expected of thematic analysis, themes did not stand alone in these complex scenarios. Rather, they were entangled with one another. For example, one respondent felt the elephant receiving taction was “enjoying a belly scratch from a trusted keeper.” This quote reflects themes of both enjoyment (of the belly scratch) and ECRs (as the elephant is said to trust the keeper). Particularly timely and of keen interest (as it is a novel finding in zoo-related studies) was the generated theme of the elephants’ engagement in labor. Though this research generated four key themes, this article focuses on the interconnected themes of elephant labor (due to the aforementioned lack of extant literature on the subject) and autonomy (due to the importance of this theme to elephant welfare, standards of ethical treatment of animals in research (Table 1), and the complimentary alignment of autonomy and humane labor for animals (detailed further in Section 3.3 and Section 3.4)).

### 3.3. Elephant Autonomy

Many respondents expressed that the elephants demonstrated decision making or a level of autonomy/agency. The theme of autonomy in this study includes references to elephants making decisions or having a choice in the activities, including elephants cooperating with their caregivers (as this too involves choice in a protected contact context). This is a key theme for this research, not only because it was mentioned under all reward and behavior video clip conditions but because some autonomy is a key component of positive animal welfare [29,45]. Though there is ongoing debate about the level of autonomy animals need or want, it is now widely understood that “training and husbandry techniques based on positive reinforcement give animals the choice of whether or not to participate, such as whether they want to interact with keepers, enter holding areas, or undergo voluntary veterinary examinations” [46] (p. 8).

Autonomy in the rewards video category often referenced that the elephants voluntarily positioned themselves for rewards to occur. For example, “holding position to best receive more food,” “he remains at the fence voluntarily,” and “could do this elsewhere at another [water] trough but choosing the one with a keeper.” Comments in this block, however, were more elaborated upon in the taction and water clips than the food clip. One respondent stated that the elephant in the taction clip seemed happy because “they do not move from the stimulation from the handler; you can almost anthropomorphize a smile on the elephant’s face.” Another respondent felt the elephant enjoyed the water interaction because “he is in control.” This was also frequently expressed regarding the selection of the water play/bathing behavior as representing the highest welfare in the reinforcers block. This sentiment was elaborated upon by one respondent who stated:

No idea if this is a thing the elephant is doing for fun or just to clean themselves or both, but they’re clearly intentionally partaking in this activity willingly and deliberately—there is space to leave, there’s no frantic/fast/uncoordinated movements that would make me think this is anything like a stress displacement behavior or obsessive/compulsive behavior.

References to autonomy in the behaviors block also noted that the elephants were free to walk away if they chose. For example, a respondent perceived the elephant felt interested during the grandstand behavior, noting:

Look, the elephant is 20 x bigger than the keeper. It isn’t going to do anything it does not want to do. This was all good training for a reward. There was nothing in the exhibit to MAKE the elephant do those asked behaviors.

Similarly, a respondent interpreted the elephants’ emotions as relaxed during the anklet behavior:

The keepers are not trying to force the elephant to participate. There are no negative tools being used. The elephant is voluntarily letting one keeper attach something to its leg—which could otherwise signal potential danger if the elephant didn’t trust the keeper.

Cooperation with their caregiver was also a frequent aspect mentioned in relation to elephants’ choices in the behaviors category. References were made to the elephant cooperating in medical or husbandry behaviors with their caregivers and that they trusted their caregivers (particularly in reference to the anklet, as in the direct quote above).

Interestingly, there was evidence that respondents did not recognize that a variety of reinforcers are utilized in zoo animal training (in advanced programs). This was indicated by comments regarding the painting behavior, such as, “she did it with no incentives (food),” “it [sic] is willingly taking part in an activity without a reward,” and “elephant presented behavior without reinforcement.” These sample comments were made although the caregiver rewarded Ellie for the painting behavior with the blow of her whistle bridge (a conditioned reinforcer), praise (though with the audio muted, it is acknowledged that these rewards could not be heard), and a grand body-language gesture where Sue threw her hands in the air and smiled widely. The assumption that the elephant received no reward for her efforts may have bolstered the perception of autonomy and related welfare. However, the voluntary nature of all of the contexts (and perhaps the substantial containment fencing between the caregivers and elephants) may have also contributed to perceptions of autonomy, as the animal could choose to walk away. Previous studies have identified that elephants’ ability to make choices regarding participation in training sessions in zoos has resulted in lower rates of compliance and, simultaneously, higher levels of welfare [45]. This may suggest that the perceptions of participants in this study support the findings of Wemelsfelder et al. [20], who found that non-animal-professionals could interpret pigs’ body language with accuracy (see additional discussion of participants’ perceptions compared to zoo professionals in Section 3.3).

Mellor et al. [29] define animal agency as “when animals engage in voluntary, self-generated and/or goal-directed behaviors,” and it is therefore closely aligned with the definition of autonomy for this research (p. 13). This agency, it is further attested, is the central premise of Domain 4 (behavioral interactions) of the FDM and is therefore key to reflecting animals’ positive welfare under conditions of interacting with caregivers, conspecifics, or their environment [29]. The specific comments reflecting the elephants’ choices to interact and to remain engaged with (or not leave) the sessions were offered as indications of positive welfare.

### 3.4. Elephant Labor

The concept of the elephants working for payment was also one of the themes generated through the RTA process. Discourse surrounding reinforcement in exchange for behavior is applicable to the theme of elephant labor. The elephant labor theme was less prevalent than the autonomy theme, as it was generated particularly for the behaviors block. This is rational, as all labor involves behavior. It is an interesting, important, and timely discussion, however, as it relates to all other themes generated (i.e., not only may all behaviors be viewed as a form of work but all rewards as a form of payment) and is an emerging field of work (specifically that of humane animal labor, e.g., [7]) within the social sciences, though no academic discourse exists regarding zoo animals’ labor. The theme of elephant labor is defined for this study as including references to animals performing labor in the form of behaviors in exchange for expected payment in the form of rewards.

Many references were made to elephants performing behaviors to receive rewards, such as “wants to perform the activity for the food reward.” This does not imply that respondents viewed the labor as being coerced. For example, a respondent proffered, “the elephant, of course, wants reinforcement and will perform conditioned criteria for said reinforcement… it’s not being forced.” Harkening back to the comments regarding the elephant painting yet receiving no identifiable reinforcement, respondents perceived that the elephants expected food reinforcement. This was evident in several comments, such as, “the elephant knows there is a treat in the bucket and is willing to perform acts to receive it” and “the animal demonstrates a level of expectation of receiving a reinforcement after completing the request.” Some respondents more overtly referenced typical facets of employment, such as, “this looks like a trained behavior… [and] the fact that they [the elephant] put their trunk out for the expected payment kinda [sic] gives it away.” Here, the respondent directly refers to the expectation of a reward as payment for a task or job.

The concept of animal labor, as described by Cochrane [47], fits particularly well with this case study. Cochrane [47] (p. 49) argues that

…good work for animals has a three-fold basis: it provides pleasure, including through affording opportunities to use and develop skills; it allows for the exercise of animals’ agency; and it provides a context in which animals can be esteemed as valuable workers and members of the communities in which they labour.

If Cochrane’s [47] theory for good work is accepted, one could argue that the elephants within the context of this study meet the proposed criteria for engaging in “good work.” Elephants in this study were often perceived as happy and enjoying the activity and were described as experiencing pleasure. Reference was also made to the elephant learning the skill of painting.

Further, this study’s theme of autonomy is related to Cochrane’s call for animals’ expression of agency, which he defines as having wants or desires and the wish or interest to act upon their wants [47]. Respondents expressed that they felt the elephants wanted to participate in the activities and that it was their choice. It was further discussed that these choices were at times in pursuit of rewards and other times not. Finally, Cochran [47] asserts that animals should be regarded as important members of the communities in which they work. Zoos are important cultural destinations within their communities [48] and are by their very nature designed to showcase the value and importance of their animal residents and to connect that value to in situ animals for conservation aims.

Pragmatically, the popularity of elephants in zoos and, more narrowly, the popularity of backstage encounters with the elephants at ZT, where the video clips for this case study were recorded, demonstrate their importance within the community of Tampa, Florida, and arguably within the zoo itself. It can be argued that Zoo Tampa stands as a micro-community comprised of resident animals (laborers), non-resident human laborers (full-time, part-time, volunteers, vendors, and consultants) who do not live at the zoo but spend a great deal of their lives there, visitors, and non-intentionally compensated resident and non-resident animals, such as migratory birds, squirrels (who are frequently compensated via dropped food from visitors and resident animals who are willing to share their meals), and a host of other local wildlife who find the zoo an ideal permanent or temporary home.

Though there is no reference specifically to working animals in the FDM, there are references to “pushing performance animals to their physiological/physical limits” as indications of compromised welfare and having potentially high negative affective impact [29] (p. 16). These cautions are suggestive of horses, and mentions are made to aversive tools, such as whips, but they can also apply to elephants (as there is documentation of historic use of coercive strategies in more antiquated programs) [49]. This type of strenuous work, however, was not represented in this study.

Requesting one grandstand behavior could not be considered approaching Ellie’s physical or mental limits. Nonetheless, there were some indications from comments that the elephants were not enjoying their labor or were not fully engaged, such as “he knew the trick to get food” and “if she wants a treat she must comply.” This may indicate a perception by a small segment of respondents of the elephants’ emotional states as compromised and the elephants themselves as made to perform. This is an important consideration for zoos to mitigate but was not representative of most respondents.

### 3.5. Comparisons of Welfare Assessments

As a point of comparison to the respondents’ perceptions of the elephants’ welfare, I conducted a welfare assessment based on Domains 4 and 5 of the FDM. Using the graded Negative Affective Impact (NAI) assessment tool [29] (p. 16), where A = None and E = Very Severe, I rated the ZT elephants’ interactions with their caregivers as “A” based on the video clips and my historic knowledge of these caregivers with these elephants. I also utilized the graded Positive Affective Impact (PAI) tool [29] (p. 17), which identifies four levels (None, Low-level, Mid-level, and High-level) on a 4-12 scale. I rated the ZT ECIs as between Mid-level and High-level, with a score of 10 of 12. Two points were withheld due to my knowledge of the amount of time spent within a workday interacting with the elephants (personal observation). These two tools provide an overall positive assessment regarding the elephants’ welfare based on ECIs at ZT.

As an additional point of comparison, I obtained ZT elephant welfare assessments conducted by the zoo’s caregivers and veterinarian that are used for their AZA accreditation records. ZT’s welfare assessments used a 1–3, rating where 1 = Poor, 2 = Average, and 3 = Good. They also relied on the FDM for welfare assessments, though they did not utilize the NAI and PAI tools described above and there is no indication that they employed Mellor et al.’s version of the FDM for welfare assessments. Rather, they defined each of the five domains (specific to ZT) and then rated each domain for each elephant on a 1–3 scale. As their definition of the Behavior Domain does not specify elephants’ interactions with caregivers but rather focuses on elephants’ interactions with conspecifics, I have not included that parameter in their evaluations here. Specifically regarding Sdudla and Ellie, the two elephant participants in this study, ZT professionals rated them both as having 3-level (good) mental (affective state) welfare. Therefore, this study suggests that the zoo-supportive participants in this research perceived elephant welfare (based on behavioral interactions and their affective states) similarly to professional animal care staff who work directly with the animals (caregivers, veterinarians, and behavioral consultants).

## 4. Conclusions

This case study sought to determine zoo supporters’ perceptions of elephants’ emotions and welfare states under conditions of elephants receiving rewards and demonstrating behaviors within the context of ECIs in a zoo to generate key themes associated with those perceptions and to compare those perceptions to zoo professionals’ through the lens of The Five Domains animal welfare assessment model [29]. This article explores two themes that were generated regarding participants’ perceptions: elephants demonstrating a level of autonomy and elephants as laborers/workers. The discourse of elephant labor in the zoo is timely, as animal labor and humane labor in particular are growing topics in social sciences research. As this is the first study to discuss this strand of animal labor, it makes a unique contribution to current literature on the topic. As evidenced by the discussion above, most respondents’ emotional descriptors and comments, filtered through the lens of the recent FDM, are indicative of perceived positive welfare.

Pragmatically, this research adds to the body of knowledge that zoos must consider as they formulate visitor experiences, largely consisting of animal–caregiver interactions in the context of training sessions. It indicates that supporters of zoos perceive ECIs as mostly positive but provides insight into areas where zoos can leave the best impression on their visitors: by highlighting the autonomous/voluntary nature of the interactions and by giving animals an increasing ability to make choices in what may be perceived by visitors as animal labor. Zoo consumers’ perceptions strongly aligned with zoo professionals’ perceptions/ratings (those of the author and the elephants’ caregiver and veterinary teams). This provides further evidence that zoo visitors may be a viable tool in the welfare evaluations of zoo animals, supporting the findings of Lacinak [3], who researched visitor perceptions involving species other than elephants. This may serve as a further point of visitor satisfaction, as it prompts visitors to contribute directly to the animals’ welfare, strengthening their role within the zoo’s micro-community. Furthermore, this study may serve as an inception point to research animal labor in zoos with a variety of species in the future, as this is an unexplored realm.

## 5. Limitations

This pool of participants was sought through college animal-oriented programs and through the author’s social media connections, which are likely to be animal-friendly. This is appropriate for this study, which seeks to understand perceptions of zoo visitors and/or supporters (as the key demographic), but it is of interest for future studies to look specifically at pools of participants outside of zoo users. Due to the acknowledged biased nature of this research (seeking to understand zoo users), these data cannot be assumed to represent non-zoo users equally.

Like many qualitative studies, this research lacks what is traditionally referred to as generalizability. Instead, it provides a level of transferability. In transferability, the reader of the work must determine if the research conducted, illustrated through thick description, “overlaps with their own situation and/or they can intuitively transfer the findings to their own action” [50] (p. 141). If so, generalization through a process of transferability can be said to be accurate.

Additionally, this case study centered on three specific rewards and three specific behaviors. Though, in my experience working in zoos, there could be more variety utilized to reinforce animals, this study represents those most frequently used. There are many more options that could be explored. This same limitation is true of behaviors; this study represents a small sample of the full repertoire of most elephant programs in accredited American zoos.

There are many aspects of elephant labor that this study illuminates as potential future research that were beyond the scope of this initial contribution to the extant literature. For example, many of the metrics associated with labor, such as cortisol or dopamine indicators related to workload or specific tasks, avoidance and/or engagement behaviors associated with labor times or precursors, risks posed to animals either physically (specific behaviors) or psychologically (coercive training methods), and other parameters, would be of interest.

## Figures and Tables

**Table 1:** Ethics and consent principles as defined by Van Patter and Blattner [13] for studies involving animal participants and this study’s practical application of each recommendation.

Principle	Definition	Practical Application
Non-Maleficence	…no animal should be harmed in research for the benefit of humans, other animals, or broader taxa, and all possible measures should be taken to understand, prevent, and mitigate any potential harms that may result from research (p. 174).	Video clips obtained for use in this research were gathered during typical daily enrichment learning sessions for the elephants based on positive reinforcement training (PRT) utilizing a reciprocal ethos of elephant–caregiver interactions. None of the behaviors requested of the elephants or rewards given to the elephants were sources of visible discomfort, and the elephants eagerly engaged.
Beneficence	…requires that animal participants benefit from research (p. 176).	The pragmatic epistemology of this research requires an actionable real-world outcome, and thus recommendations based on data analysis that may directly benefit zoo elephants are proposed. Further, the welfare analysis conducted serves to evaluate the benefits of PRT ECI activities at Zoo Tampa (ZT) and may be extrapolated to other zoos.
Voluntary Participation	…animals cannot be forced to participate in research (p. 179).	Elephants were called by a caregiver individually to participate in a PRT learning session, and their approach was interpreted as consent. Nutrition/diets (inducements) were not withheld at any point of the video gathering process, and no coercive tools or other aversive methods were employed.

**Table 2:** Simple summary of Clips A-F. * indicates selection by participants as reflecting the highest welfare of the clips in each category.

Category	Clip Label	Description	Participants’ Descriptors of Elephants’ Emotions	Link for Clip
Rewards	A	Produce	happy, content, hungry, excited	https://youtu.be/q4Nv8yRrvTQ (accessed on 23 November 2025)
	B	Taction	relaxed, content, comfortable, happy	https://youtu.be/4zk7tGh0yUg (accessed on 23 November 2025)
	C	Water *	hot, warm, happy, enjoying, playful	https://youtu.be/qeY5B39a2AU (accessed on 23 November 2025)
Behaviors	D	Grandstand	engaged, motivated, interested	https://youtu.be/Vc095f1Tx5Y (accessed on 23 November 2025)
	E	Painting	happy, engaged, interested, stimulated	https://youtu.be/B9z3vEncLMs (accessed on 23 November 2025)
	F	Anklet *	trusting, calm, relaxed, patient	https://youtu.be/ynHieb5gRYc (accessed on 23 November 2025)

**Table 3:** Sample of RTA coding and thematic mapping for this case study.

Sample Quotes	Codes Assigned	Themes Defined
they do not move from the stimulation from the handler; you can almost anthropomorphize a smile on the elephant’s face	proximity, body language, choice, relationship, enjoyment	Autonomy: includes references to elephants making decisions or having a choice in the activities, including cooperating with their caregivers (themes of ECR and enjoyment not explored and therefore not defined in this publication).
this looks like a trained behavior… [and] the fact that they [the elephant] put their trunk out for the expected payment kinda [sic] gives it away	PRT, body language, payment/reward, work/labor	Labor: includes references to animals performing labor in the form of behaviors in exchange for expected payment in the form of rewards.

## Data Availability

The data that support the findings of this study are available from the corresponding author upon reasonable request.

## Abbreviations

The following abbreviations are used in this manuscript:

APHIS	Animal and Plant Health Inspection Service
AZA	Association of Zoos and Aquariums
ECI	Elephant–caregiver interaction
FDM	Five Domains model
OSHA	Occupational Safety and Health Association
PRT	Positive reinforcement training
USDA	United States Department of Agriculture
ZT	Zoo Tampa at Lowry Park

## References

[1] Carr N. (2016). An analysis of zoo visitors’ favourite and least favourite animals. Tour. Manag. Perspect..

[2] Fernandez E.J., Martin A.L. (2021). Animal training, environmental enrichment, and animal welfare: A history of behavior analysis in zoos. J. Zool. Bot. Gard..

[3] Lacinak A.M. (2023). Animals’ relationships with caregivers and conspecifics are associated with zoo visitors’ perceptions of animals’ emotions and welfare states. Anthrozoös.

[4] Lacinak A.M. (2024). Zoo visitors’ most-liked aspects of elephant encounters and related perceptions of animals’ emotions and welfare states: A pragmatic approach. Animals.

[5] AZA—Association of Zoos and Aquariums (2025). 2026 Accreditation Standards & Related Policies. https://assets.speakcdn.com/assets/2332/accred_standards.pdf.

[6] Grazian D. (2015). American Zoo: A Sociological Safari.

[7] Blattner C.E., Coulter K., Kymlicka W. (2020). Animal Labour: A New Frontier of Interspecies Justice?.

[8] Coulter K. (2016). Animals, Work, and the Promise of Interspecies Solidarity.

[9] Coulter K. (2016). Beyond human to humane: A multispecies analysis of care work, its repression, and its potential. Stud. Soc. Justice.

[10] Coulter K. (2017). Humane jobs: A political economic vision for interspecies solidarity and human-animal wellbeing. Politics Anim..

[11] Coulter K., Blattner C., Coulter K., Kymlicka W. (2020). Toward humane jobs and work-lives for animals. Animal Labour: A New Frontier of Interspecies Justice?.

[12] Ward S., Sherwen S., Hosey G., Melfi V. (2019). Zoo animals. Anthrozoology: Human-Animal Interactions in Domesticated and wild Animals.

[13] Van Patter L.E., Blattner C. (2020). Advancing ethical principles for non-invasive. respectful research with nonhuman animal participants. Soc. Anim..

[14] Kelly L.M., Cordeiro M. (2020). Three principles of pragmatism for research on organizational processes. Methodol. Innov..

[15] Wassermann S.N., Hind-Ozan E.J., Seaman J. (2018). Reassessing public opinion of captive cetacean attractions with a photo elicitation survey. PeerJ.

[16] Griffin T. (2019). A discussion of video as a data collection tool. Curr. Issues Tour..

[17] Greco B.J., Meehan C.L., Miller L.J., Shepherdson D.J., Morfeld K.A., Andrews J., Baker A.M., Carlstead K., Mench J.A. (2016). Elephant management in North American zoos: Environmental enrichment, feeding, exercise, and training. PLoS ONE.

[18] Nayak M.S.D.P., Narayan K.A. (2019). Strengths and weaknesses of online surveys. IOSR J. Humanit. Soc. Sci..

[19] Ruel E., Wagner W.E., Gillespie B.J. (2016). The Practice of Survey Research: Theory and Applications.

[20] Wemelsfelder F., Hunter T.E.A., Mendl M.T., Lawrence A.B. (2001). Assessing the ‘whole animal’: A free choice profiling approach. Anim. Behav..

[21] Minero M., Costa E.D., Dai F., Murray L.A.M., Canali E., Wemelsfelder F. (2016). Use of qualitative behaviour assessment as an indicator of welfare in donkeys. Appl. Anim. Behav. Sci..

[22] Binding S., Farmer H., Krusin L., Cronin K. (2020). Status of animal welfare research in zoos and aquariums: Where are we, where to next?. J. Zoo Aquar. Res..

[23] Mellor D.J., Stafford K.J. (2001). Integrating practical, regulatory and ethical strategies for enhancing farm animal welfare. Aust. Vet. J..

[24] Mellor D.J. (2004). Comprehensive assessment of harms caused by experimental, teaching and testing procedures on live animals. Altern. Lab. Anim..

[25] Mellor D.J., Patterson-Kane E., Stafford K.J. (2009). The Sciences of Animal Welfare.

[26] Mellor D.J. (2012). Affective states and the assessment of laboratory-induced animal welfare impacts. ALTEX Proc..

[27] Mellor D.J., Beausoleil N.J. (2015). Extending the “Five Freedoms” model for animal welfare assessment to incorporate positive welfare states. Anim. Welf..

[28] Mellor D.J. (2017). Operational details of the Five Domains Model and its key applications to the assessment and management of animal welfare. Animals.

[29] Mellor D.J., Beausoleil N.J., Littlewood K.E., McLean A.N., McGreevy P.D., Jones B., Wilkins C. (2020). The 2020 Five Domains Model: Including human-animal interactions in assessments of animal welfare. Animals.

[30] Mellor D.J., Hunt S., Gusset M. (2015). Caring for Wildlife: The World Zoo and Aquarium Animal Welfare Strategy.

[31] Marton B., Kilbane T., Nelson-Becker H. (2019). Exploring loss and disenfranchised grief of animal care workers. Death Stud..

[32] Obilor E.I. (2023). Convenience and purposive sampling techniques: Are they the same?. Int. J. Innov. Soc. Sci. Educ. Res..

[33] Lindemann N. (2021). What’s the Average Survey Response Rate?. https://pointerpro.com/blog/average-survey-response-rate/.

[34] Yilmaz S., Duzenli T., Cigdem A. (2017). Visitors experiences in different zoo exhibits. Curr. World Environ..

[35] Lukas K.E., Ross S.R. (2014). Naturalistic exhibits may be more effective than traditional exhibits at improving zoo-visitor attitudes toward African apes’. Anthrozoös.

[36] Miller L.J., Luebke J.F., Matiasek J. (2018). Viewing African and Asian elephants at accredited zoological institutions: Conservation intent and perceptions of animal welfare. Zoo Biol..

[37] Millwood-Lacinak A. (2018). Factors That Influence African Elephant (Loxodonta Africana) Participation in Positive Reinforcement Learning Contexts. Master’s Thesis.

[38] Razal C.B., Miller L.J. (2019). Examining the impact of naturalistic and unnaturalistic environmental enrichment on visitor perception of naturalness, animal welfare, and conservation. Anthrozoös.

[39] Braun V., Clarke V. (2022). Thematic Analysis: A Practical Guide.

[40] Braun V., Clarke V. (2021). Can I use TA? Should I use TA? Should I not use TA? Comparing reflexive thematic analysis and other pattern-based qualitative analytic approaches. Couns. Psychother. Res..

[41] Braun V., Clarke V. (2021). One size fits all? What counts as quality practice in (reflexive) thematic analysis?. Qual. Res. Psychol..

[42] Braun V., Clarke V. (2019). To saturate or not to saturate? Questioning data saturation as a useful concept for thematic analysis and sample-size rationales. Qual. Res. Sport Exerc. Health.

[43] De Waal F. (2020). Interviewed by Angela M. Lacinak.

[44] Burns M. (2019). Interviewed by Angela Lacinak, Zoo Tampa.

[45] Wilson M., Perdue B.M., Bloomsmith M.A., Maple T.L. (2015). Rates of reinforcement and measures of compliance in free and protected contact elephant management systems. Zoo Biol..

[46] Browning H., Veit W. (2021). Freedom and animal welfare. Animals.

[47] Cochrane A., Blattner C., Coulter K., Kymlicka W. (2020). Good work for animals. Animal Labour: A New Frontier of Interspecies Justice?.

[48] Fraser J., Switzer T. (2021). The Social Value of Zoos.

[49] Brando S., Norman M. (2023). Handling and training of wild animals: Evidence and ethics-based approaches and best practices in the modern zoo. Animals.

[50] Smith B. (2018). Generalizability in qualitative research: Misunderstandings, opportunities and recommendations for the sport and exercise sciences. Qual. Res. Sport Exerc. Health.

